# Prevalence and Diagnosis of PCOS Using Electronic Health Records: A Scoping Review and a Database Analysis

**DOI:** 10.3390/ijerph21030354

**Published:** 2024-03-15

**Authors:** William Atiomo, Mohamed Nor Haq Rizwan, Muhammad Hamza Bajwa, Hussain Juzer Furniturewala, Komal Sundeep Hazari, Deemah Harab, Widad Abdelkareem, Sumayya Inuwa, Amar Hassan Khamis, Muna Tahlak, Fadi G. Mirza

**Affiliations:** 1 Department of Clinical Sciences, College of Medicine, Mohammed Bin Rashid University of Medicine and Health Sciences, Building 14, Dubai Healthcare City, Dubai P.O. Box 505055, United Arab Emirates; mohamednorhaq.rizwan@students.mbru.ac.ae (M.N.H.R.); muhammad.bajwa@students.mbru.ac.ae (M.H.B.); hussain.furniturewala@students.mbru.ac.ae (H.J.F.); sumayya_inuwa@hotmail.com (S.I.); amar.hassan@mbru.ac.ae (A.H.K.); 2 Latifa Women and Children Hospital, Dubai P.O. Box 9115, United Arab Emirates; khazari@dubaihealth.ae (K.S.H.); dkharab@dubaihealth.ae (D.H.); waabdelkareem@dubaihealth.ae (W.A.); matahlak@dubaihealth.ae (M.T.); fgmirza@dubaihealth.ae (F.G.M.)

**Keywords:** polycystic, ovary, syndrome, ICD, diagnosis, scoping, review, electronic, health, records

## Abstract

Polycystic ovary syndrome (PCOS) is the most common endocrine disorder affecting women of reproductive age. It increases the risk of type 2 diabetes, cardiovascular disease, endometrial cancer, infertility, gestational diabetes, preeclampsia, and preterm birth. Accurately identifying predictors of these health risks is crucial. Electronic health records (EHRs) offer an affordable approach, however, the validity and reliability of EHRs for PCOS diagnosis are unclear. A scoping review of the literature on the prevalence and reliability of the diagnosis of PCOS using EHRs was performed. An analysis of the feasibility of obtaining diagnostic variables from a PCOS patient database was also carried out. Eight studies met the criteria. The prevalence of PCOS ranged from 0.27% to 5.8%. Reliability varied, with one study reporting a sensitivity of 50% and a specificity of 29%. Another study found a 74.4% agreement between international classification of disease (ICD) codes and clinical criteria. The database analysis found only 13.7%, 8%, and 7.5% of women had all the necessary variables for an objective diagnosis of PCOS using the Rotterdam, National Institutes of Health (NIH), and Androgen Excess and PCOS Society (AEPCOS) criteria, respectively. Using EHRs results in an underestimation of PCOS prevalence compared to other diagnostic criteria, and many women identified may not meet the complete diagnostic criteria. These findings have implications for future research studies on PCOS prevalence and related health risks.

## 1. Introduction

Polycystic ovary syndrome (PCOS), a prevalent and complex endocrine disorder, affects approximately 21% of women of reproductive age [1]. It is characterized by hyperandrogenism, ovulatory dysfunction, and polycystic ovarian morphology, leading to various reproductive, metabolic, and psychological issues [2]. Additionally, PCOS is linked to a higher risk of type 2 diabetes, cardiovascular disease, endometrial cancer, infertility, and pregnancy complications [3]. Research is needed to identify predictors of these risks in women with PCOS to reduce the associated morbidity and mortality. However, diagnosing PCOS for large epidemiological studies can be challenging and costly due to the need for specific tests, the variability in clinical features, and the lack of a standardized diagnostic method. Cultural barriers could present challenges in certain situations, such as the requirement of trans-vaginal ultrasound scans for women who are not sexually active or uncomfortable with the procedure. Utilizing electronic health records (EHRs) may offer a more accessible and cost-effective method for addressing research inquiries in large epidemiological studies on PCOS.

Multiple diagnostic criteria have been suggested for diagnosing PCOS over time, including the National Institutes of Health (NIH) criteria, the Rotterdam criteria [4], the Androgen Excess and PCOS Society (AEPCOS) criteria [5], and the International Evidence-based Guideline for the Assessment and Management of PCOS [6]. Despite the variety of criteria available, there is no agreement on the most suitable or precise criteria for diagnosing PCOS. Additionally, different criteria may lead to the identification of distinct subgroups of women with PCOS [7], complicating the use of EHRs in identifying the condition in large epidemiological studies.

International Classification of Diseases (ICD) codes are widely used to identify and classify diseases in EHRs, which are increasingly utilized for epidemiological research, health services evaluation, and quality improvement [8]. However, the validity and reliability of ICD codes for diagnosing PCOS are unclear. Previous studies found that it may be difficult to use codified data from EHRs and confidently identify PCOS. In one publication, 13–20% of adolescents with PCOS were misclassified when using the ICD, 9th edition code (ICD-9) as an identifier [9]. Conversely, a separate study found that only 73% of adolescents were confirmed to have a diagnosis of PCOS [10]. Among adult women diagnosed with PCOS using an ICD-9 code [11], only 28% had documented anovulation and clinical hyperandrogenism in their records, while an additional 52% had only one of these features documented.

Using ICD codes for diagnosing PCOS presents another challenge, as the availability and completeness of diagnostic variables in electronic health records (EHRs) may be limited. The Rotterdam criteria, the most commonly used for diagnosing PCOS, necessitate the presence of at least two of the following: irregular ovulation, increased androgen levels, and polycystic ovaries on ultrasound [4]. However, not all of these variables may be documented in EHRs, particularly for women not undergoing fertility treatment or with mild symptoms. This raises questions about the accuracy and practicality of confirming a PCOS diagnosis using ICD codes in EHRs. Previous literature reviews [12,13] explored this uncertainty on the accuracy of EHRs in the diagnosis of other health conditions, but we were not able to identify any previously published reviews on PCOS.

The aim of this study was to conduct a scoping review of the literature on the prevalence and reliability of diagnosing PCOS with EHRs and to perform a feasibility analysis of the availability of the diagnostic variables in a database of PCOS patients. We hypothesized that the reliability of diagnosing PCOS based on EHRs would be poor to moderate and that the proportion of women who had the required variables recorded to validate an ICD diagnosis of PCOS using the Rotterdam criteria would be low in the database of PCOS patients.

## 2. Materials and Methods

Institutional review board approval was not required for the scoping review because it was a secondary review of primary studies in the literature, and we did not have direct contact with patients. For the database analysis, the Dubai Scientific Research Ethics committee (DSREC) granted institutional review board approval for this study (Reference number, DSREC-09/2022_21) in September 2022.

### 2.1. Scoping Review

We conducted a scoping review following the methodological framework outlined by Arksey and O’Malley [14] as well as utilizing the PRISMA-ScR checklist [15]. Our search was carried out in the databases PubMed, Scopus, and Cochrane. For studies which assessed the reliability of the diagnosis of PCOS, using EHRs compared to other diagnostic features we used the following search terms: “Electronic Health Records AND Polycystic Ovar* Syndrome OR Polycystic Ovar* Disease”, “Diagnosis AND PCO*” and “ICD Diagnosis of PCOS AND Irregular menstrual cycles”. Reference lists of the initially identified studies were reviewed, and relevant secondary references were included. We limited the search to studies published in English from the inception of available literature until June 2023. Three authors (M.N.H.R., M.H.B., and H.J.F) independently screened the titles of the identified studies. Any discrepancies that arose during this initial screening were resolved through in-depth discussion and consensus-building among the authors. Subsequently, studies that passed this initial title screening underwent a full-text retrieval and review.

Data extraction from the selected studies was carried out by entering data into a structured summary table, with categories encompassing prevalence (%) of PCOS, diagnostic criteria employed in the study, sample size, age group of the study population, first author, country or region where the study was conducted, year of publication, and whether the studies assessed the reliability of the EHR diagnosis of PCOS.

### 2.2. Database Analysis

We requested a report from the Dubai Health Authority (DHA) electronic patient medical record system, Salama to determine if the 1031 women identified in our previous study [16] had the variables required for an objective diagnosis of PCOS using the Rotterdam, NIH, and AEPCOS criteria recorded in the database. The Salama system is available at a variety of DHA health-care facilities [16]. The system is seamlessly integrated with over 30 key clinical and administrative systems of DHA, which include radiology, laboratory, endocrinology, and cardiology, as well as Dubai ambulance services. This system houses more than 5 million patient medical records. On 30 May 2023, an Excel spreadsheet containing the medical record numbers of 1031 patients diagnosed with PCOS in our original study [16] was provided to the Salama database administration team. A report was requested for each patient in the spreadsheet to determine if their information was recorded in the Salama database, marked as either “yes” or “no”.

The clinical variables included the following, oligomenorrhea (ICD code N91/N91.2/N92.5/N92.6), hirsutism (ICD code L68.0), hair loss or alopecia (ICD code L64/L65), and female infertility associated with anovulation (ICD code N97.0). As well as recording whether the clinical variables outlined above were recorded in the Salama database for each patient, based on the presence or absence of the relevant ICD codes as outlined above, we checked if the patients had had the following investigations performed: pelvic ultrasound and certain blood tests—serum testosterone, sex hormone binding globulin (SHBG), luteinizing hormone (LH), follicle stimulating hormone (FSH), prolactin (PL), thyroid function test (TFT), and 17-OH progesterone (17-OH-P).

We then determined how many of the 1031 women classed as PCOS using the ICD codes in our previous study, had the variables required to make an objective diagnosis using the Rotterdam [4], NIH [4], and Androgen excess and PCOS (AEPCOS) [17] society criteria.

The Rotterdam consensus defines polycystic ovarian syndrome (PCOS) as the presence of two of three of the following features: oligo-anovulation, hyperandrogenism polycystic ovaries on ultrasound, and exclusion of other causes of chronic oligo or anovulation. We started with the Rotterdam criteria because it is the broadest classification of PCOS currently in use. The populated Excel spreadsheet was then analyzed to determine the proportion of women who had a “yes” in two of three of the following fields.

1. “Yes” in at least one of oligomenorrhea (ICD code N91/N91.2/N92.5/N92.6) or female infertility associated with anovulation (ICD code N97.0) as evidence of chronic oligo-anovulation.
2. “Yes” in at least one of hirsutism (ICD code L68.0), hair loss or alopecia (ICD code L64/L65), or having had a serum testosterone conducted as evidence of hyperandrogenism.
3. “Yes” to having had a pelvic ultrasound scan carried out as evidence that polycystic ovaries with ultrasound had been assessed.

AND “Yes” to having had all of the following endocrine investigations conducted: LH, FSH, PL, and TFT as evidence that other causes of chronic oligo or anovulation had been assessed.

The NIH criteria for the diagnosis of PCOS, requires the presence of all the following: clinical or biochemical evidence of hyperandrogenism, chronic anovulation, and exclusion of other known disorders.

The populated Excel spreadsheet was therefore analyzed to determine the proportion of women who had a “yes” in all of the following fields.

1. “Yes” in at least one of hirsutism (ICD code L68.0), hair loss or alopecia (ICD code L64/L65), or having had a serum testosterone (with or without sex hormone binding globulin (SHBG)) carried out as evidence of hyperandrogenism.
2. “Yes” in at least one of oligomenorrhea (ICD code N91/N91.2/N92.5/N92.6) or female infertility associated with anovulation (ICD code N97.0) as evidence of chronic anovulation.
3. “Yes” to having had all the following endocrine investigations performed: LH, FSH, PL, TFT, and 17-hydroxy progesterone (17-OH P) as evidence that other known disorders had been excluded.

The AEPCOS criteria requires the diagnostic criteria for PCOS to include all of the following: hyperandrogenism (hirsutism and/or hyperandrogenemia), ovarian dysfunction (oligo-anovulation and/or polycystic ovaries), and exclusion of other androgen excess or related disorders.

The populated Excel spreadsheet was analyzed to determine the proportion of women who had a “yes” in all of the following fields.

1. “Yes” in at least one of hirsutism (ICD code L68.0), hair loss or alopecia (ICD code L64/L65), or having had a serum testosterone (with or without sex hormone binding globulin (SHBG)) performed as evidence of hyperandrogenism.
2. “Yes” in at least one of oligomenorrhea (ICD code N91/N91.2/N92.5/N92.6) or female infertility associated with anovulation (ICD code N97.0) as evidence of chronic oligo-anovulation and/or “Yes” to having had a pelvic ultrasound scan carried out as evidence of ovarian dysfunction.
3. “Yes” to having had all of the following endocrine investigations conducted: LH, FSH, PL, TFT, and 17-hydroxy progesterone (17-OH P) as evidence that other androgen excess or related disorders had been excluded.

### 2.3. Statistical Analysis

Variables were summarized as categorical variables and the proportion of the 1031 women who had had a pelvic ultrasound scan, oligo-anovulation, hyperandrogenism, and all the required endocrine assays to exclude other causes of chronic oligo or anovulation was reported as proportions separately. Finally, the proportion of women who had all the required variables recorded on the Salama database to enable an objective diagnosis of PCOS based on the Rotterdam, NIH, and AEPCOS criteria was calculated. As the diagnostic criteria for PCOS in adolescents differs from adults, we also separated the two age groups in our scoping review and database analysis.

## 3. Results

### 3.1. Scoping Review

**Figure 1:**
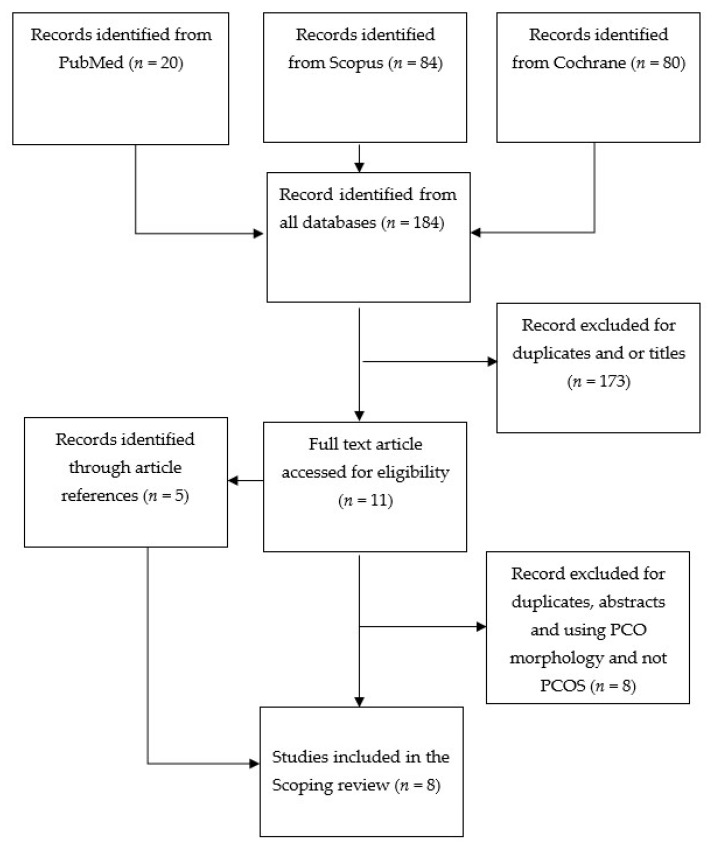
PRISMA flowchart.

The PRISMA flow chart (Figure 1) summarizes the data collection process. A total of 184 studies were identified during this search. After excluding studies that did not meet the eligibility criteria and duplicates, eight papers [10,16,18,19,20,21,22,23] were identified which were included in the final scoping review (Table 1).

The studies included (Table 1), were published between 2006 and 2023. There were seven cross-sectional studies, with one of these [23] extracted from a systematic review and meta-analysis [24], and one retrospective observational study. Geographically, the studies were conducted in different regions, including five in the United States of America, one in Europe, one in the United Arab Emirates and one in Oman. The racial demographics varied across the studies, with the studies conducted in the United States of America including multi-ethnic populations, while the studies in the United Arab Emirates and Oman mainly focused on the local population. The European study [22] encompassed all countries in Europe. The age groups included in the studies ranged from 15 years to 45 years, with a specific study [10] focusing exclusively on adolescents aged 15 to 19 years. The sample sizes of the primary studies varied, from 3644 to 12,171,830 women [24]. Not all studies in the scoping review provided the prevalence of PCOS in adolescents. Even in the studies that separated prevalence by age, it was often conducted in the 15–19-year age group. The prevalence of PCOS in the 15–19-year age group in these studies was 0.81% [21], 0.56% [10], and 0.73% [23].

The prevalence of PCOS based on EHRs across the eight studies ranged from 0.27% to 5.8%. The lower end of the prevalence spectrum was found in a retrospective observational study [22] that analyzed data from 1990 to 2016 across Europe, resulting in a prevalence of 0.27%. A study [10] conducted in the United States on adolescent females reported a prevalence of 0.56%, which increased to 1.14% when considering undiagnosed cases with documented symptoms. Another study [23] aimed to investigate the prevalence of PCOS based on a diagnosis made using the ICD code, in clinical practice, using Medicaid data from Louisiana, in the USA. Of 259,904 women who met the inclusion criteria, 2284 (0.88%) had a diagnosis of PCOS or oligomenorrhea/amenorrhea plus hirsutism. On the upper end, a cross sectional study from the USA [18] found a prevalence of 5.8% using ICD-9 and ICD-10 codes and the presence of a PCOS-related keyword to define PCOS [10].

Two studies [10,18] verified the reliability of EHRs in diagnosing PCOS within the same cohort. One study [18] compared different algorithms for identifying PCOS cases and reported sensitivity and specificity values. The study employed an algorithm named “keyword strict”, which only classified patients as having PCOS if they met the ICD code criteria for PCOS plus an additional PCOS-related keyword in their medical records. These keywords included “polycystic ovaries”, “PCOS”, or “PCO” in a clinical note. The prevalence from these cases was compared to the gold standard diagnostic criteria for PCOS to determine the sensitivity of the PCOS prevalence finding. The study reported a sensitivity of 50% and a specificity of 29%. The study also noted that changing the code from “keyword strict” to “coded-broad”, which defined cases as having PCOS if they possessed only one of the Rotterdam criteria, increased the prevalence as it included subclinical cases. However, the study did not assess the sensitivity of these findings against a gold standard diagnosis.

The second study [10] compared the prevalence obtained through ICD codes to that obtained from the NIH criteria. Of the 1055 patients identified as having PCOS through ICD codes, only 774 were confirmed as having PCOS when validated against the NIH criteria, demonstrating a 73.4% agreement. The study also investigated unidentified cases in the sample population and evaluated them based on the NIH criteria. This search revealed that an additional 789 patients met the NIH criteria for PCOS but were not coded as having PCOS using ICD codes. This meant an increase in prevalence from 0.5% to 1.03%. Overall, the study found that changing the inclusion criteria, for having a diagnosis of PCOS from ICD codes alone to ICD codes plus NIH-identified cases, increased the prevalence from 0.56% to 1.14%.

The prevalence of PCOS diagnosed through EHRs was compared with that diagnosed through other criteria in independent cohorts, in two additional studies [16,24]. The first study [16] found a prevalence of 1.60% through EHR-based identification. The study observed that the expected prevalence rates in the population would be around 17.4% and 9.45% if the Rotterdam criteria or NIH criteria were used, respectively. Additionally, it also stated that if self-reported diagnosis were to be used as an inclusion criterion for PCOS positivity, the expected prevalence would be around 19.45%. The other study [24], was a systematic review and meta-analysis that found a prevalence of 0.88% in the one study where the ICD-9 was used to define PCOS. When compared to the expected prevalence through other criteria, it was found that the prevalence would be expected to be around 16% using the Rotterdam criteria and 12% using the Androgen Excess and PCOS Society (AEPCOS) criteria.

Overall, this scoping review provided an overview of the general characteristics of the studies included and the prevalence of PCOS based on EHRs. It also emphasized the need for further investigation into the reliability of EHRs in diagnosing PCOS and highlighted the potential underestimation of prevalence by EHRs compared to other diagnostic criteria.

### 3.2. Database Analysis

In our previous study [16] we discovered a period prevalence of PCOS of 1.6% among 64,722 women aged between 15 and 45 years, by utilizing the ICD-10 codes recorded in EHRs. These consisted of 1031 of these women, who were coded as having a PCOD/PCOS diagnosis in the period from 22 August 2017 to 29 December 2022. The ICD-10 codes E28.2 and Z87.42 were used to identify women with polycystic ovary syndrome and the free text under the ICD code E28.2 including any of the following: bilateral polycystic ovarian syndrome, PCO (polycystic ovaries), PCOD (polycystic ovarian disease), PCOS (polycystic ovarian syndrome), polycystic disease, ovaries, polycystic ovarian disease, polycystic ovarian syndrome, polycystic ovaries, polycystic ovary disease, polycystic ovary syndrome and polycystic ovary. Women with the Z87.42 ICD-10 code, where the free text stated H/O polycystic ovarian syndrome, history of PCOS or history of polycystic ovaries, were also included in the polycystic ovary syndrome group. Nine hundred and sixty-three (93.4%) of the women had an ICD code of E28.2 and 68 (6.4%) had a code of Z87.42. Table 2, also described in our previous publication [16], outlines the number and proportion of women with each ICD code descriptor. The most common ICD code descriptor was PCOS (polycystic ovarian syndrome), which had a percentage of 36.9%. The ICD code descriptor PCO (polycystic ovaries) had a percentage of 20.5%. The remaining ICD code descriptors had percentages ranging from 0.1% to 9.9%.

We then determined how many of the 1031 women classed as PCOS using the ICD codes had the variables required to make an objective diagnosis using the Rotterdam criteria. Of these, 890 women (86.3%) had had an ultrasound scan carried out, and 544 women (52.8%) had “Yes” in at least one of oligomenorrhea or female infertility associated with anovulation as evidence of chronic oligo-anovulation. Of these, 248 (24%) women had “Yes” in at least one of hirsutism, hair loss or alopecia, or having had a serum testosterone performed as evidence of hyperandrogenism. Of these, 66 (6.4%) and 184 (17.8%) women had a “Yes” to having their SHBG (required to calculate the free androgen index) and 17-hydroxyprogesterone levels respectively, conducted. Of these, 647 (66.8%) of women had a “Yes” to having had all the following endocrine investigations carried out: LH, FSH, PL, and TFT as evidence that other causes of chronic oligo or anovulation had been assessed.

Of all the 1031 women in the database only 141 women (13.7%) had all the complete variables required to make an objective diagnosis of PCOS using the Rotterdam criteria, 83 women (8%) had all the complete variables required to make an objective diagnosis of PCOS using the NIH criteria, and 78 women (7.5%) had all the complete variables required to make an objective diagnosis of PCOS using the AEPCOS criteria. Percentages of 12%, 1.2% and 0.7% of women who had all the complete variables required to make an objective diagnosis of PCOS using the NIH, AEPCOS and Rotterdam criteria, respectively, were adolescents. There was however no statistically significant difference in the distribution of variables required to make a diagnosis of PCOS by age groups (Table 3).

## 4. Discussion

Obtaining a diagnosis for PCOS can pose challenges in conducting large epidemiological studies, mainly due to the expensive nature of the required tests. Additionally, cultural barriers may arise in certain situations, such as when transvaginal ultrasound scans are needed for women who are either not sexually active or uncomfortable with this procedure. Hence, the use of EHRs presents a promising and cost-effective alternative for gathering statistics on PCOS prevalence, comorbidities, and long-term health risks in large populations, offering a more user-friendly approach. However, the results of our scoping review suggested potential underestimation of the prevalence of PCOS, when coding from EHRs is used, compared to other diagnostic criteria. The database analysis, also suggests that a large portion of the women identified as having PCOS based on ICD codes following review of EHRs may not meet the complete diagnostic criteria, as only 13.7% of the women in our study had all the complete variables required for an objective diagnosis using the Rotterdam criteria. The findings that only a few studies in the scoping review separated prevalence by age group (adolescents and adults) and that the database analysis did not find any difference in the distribution of variables required to make a diagnosis of PCOS by age groups, suggest a need for increased awareness amongst practicing clinicians about the need to consider the age of the patient in deciding what diagnostic criteria for PCOS are appropriate. These findings have significant implications for future research studies on the prevalence, comorbidities and long-term health risks of PCOS, where ICD codes alone are used in defining PCOS.

The accuracy of ICD codes for the diagnosis of PCOS could have been influenced by several factors, including the proper diagnosis and interpretation of clinical symptoms, endocrine, and ultrasound findings by health care professionals, while a lack of consensus regarding the diagnostic criteria for PCOS (Rotterdam, NIH, or AEPCOS) may also result in inconsistencies in the investigations requested to make a diagnosis of PCOS. These factors pose challenges for studies like ours which seek to determine the accuracy of the coded information. In addition to the coding process, the reliability of ICD codes also depends on the documentation and reporting practices of healthcare providers. For instance, in the DHA electronic patient medical record system, Salama, there are multiple terms for the same diagnosis, making coding challenging for busy clinicians. Clinicians in busy clinics may assign codes they believe are correct, but these diagnoses may be inaccurate or subject to change once laboratory or imaging results are available. While there is an option to correct these diagnoses in Salama, it may not always be utilized.

The effectiveness of the EHR ultimately depends on human input. It only pulls information from specific documentation templates, such as the visit diagnosis and problem list. If these templates are not properly filled out and updated, the system cannot extract the information, even if it is documented in other areas like the free text patient’s note entry commonly used by physicians. Another possible reason for underreporting of PCOS in our context is insurance coverage. Unfortunately, some health insurance companies consider PCOS to be a congenital abnormality and the physicians are forced to enter another diagnosis such as insulin resistance. However, we strive to educate and encourage our physicians on the importance of utilizing the EHR to ensure accurate and easily accessible data. The EHR was implemented in the Dubai Health Authority in November 2017, and progress has been made in improving its use by training and support for physicians. We acknowledge the existing challenges, however, and work is ongoing to address them.

The ICD is the most commonly used classification system for diseases globally, with revisions overseen by the World Health Organization (WHO). The latest version, ICD-11, was approved by the WHO Assembly in May 2019 and took effect on 1 January 2022 [25]. However, previous studies have reported concerns on accuracy of the ICD codes. In one study [26] the reliability of diagnoses coding with the ICD-10 in a national healthcare system was compared between the following three groups: students, medical managers, and coding specialists. The results found that agreement in principal diagnosis was fair to moderate for terminal codes and substantial for the chapter level. However, the results were lower than in previous publications, indicating significant uncertainties even for experts. The study concluded that the complexity of the ICD-10 required simplification to obtain an accurate representation of the patient’s diagnosis and make coded data useful for quality management, healthcare financing, and healthcare policy. In another study [27], sources of errors in the inpatient ICD coding process were examined. The study identified various sources of errors at each step of the coding process, including information at admission, communication among healthcare providers, clinician’s knowledge and experience, and electronic and written records. In our opinion, ensuring that ICD codes accurately reflect the diagnosis of PCOS using objective criteria mainly requires that the templates, from which coders and EHR coding databases pull their information to code the diagnosis, are properly completed by the healthcare professionals who see the patients in the healthcare clinics/facilities. More awareness amongst healthcare professionals about the importance of using objective criteria for diagnosis of PCOS might also help address this matter.

Within the context of PCOS, these challenges in the reliability of its ICD coding are also increased by recently (2018) updated international diagnostic criteria. In a study [28] investigating the new 2018 guidelines for the diagnosis, only 76% of women with PCOS based on the Rotterdam criteria met the criteria based on the new 2018 guidelines. Furthermore, in studies investigating comorbidities of PCOS, when the diagnosis was made based on ICD codes, challenges were encountered. For example, in a study [29] which examined data from the National Health Care Surveys to understand patient demographics and behavioral health services associated with ICD coded PCOS-related medical visits, the study found that data on mental health and health education did not meet the necessary criteria for reliable national estimates. Unfortunately, it is unlikely that recent attempts to improve the international federation of gynecology and obstetrics (FIGO) classification of ovulatory disorders [30] will address these challenges as even though the new classification now includes ovulatory disorders categorized into four groups, with a new stand-alone category for PCOS, in the development process using a Delphi approach, no consensus was reached on the question of using the Rotterdam criteria for the diagnosis of PCOS.

The prevalence of PCOS varies according to ethnic background. In 2019, Ganie et al. [31] found that the prevalence of PCOS in India ranged from 3.7–22.5%. A meta-analysis conducted by Deswal et al. [1] found that Southern China, Iran, and the USA reported a prevalence of 2.2%, 3%, and 4%, respectively. Beijing, Palestine, Brazil, Sri Lanka, the UK, Greek, and Spain found a prevalence rate in the range of 5–10%, whilst Australia, Turkey, and Denmark reported a higher prevalence of 15–20%. Relying on EHRs, therefore, appears to underestimate the prevalence of PCOS regardless of ethnicity, as found in our scoping review, where the prevalence of PCOS based on ICD codes ranged from 0.27% to 5.8% at most. The implications of this means that PCOS may be underdiagnosed, particularly in certain ethnic groups while healthcare providers should be cautious when relying solely on EHR data for diagnosis and research. Future studies examining the accuracy and reliability of EHRs in capturing the prevalence of PCOS, particularly in diverse populations, would also be beneficial.

The strengths of our study are in its novelty, as we are unaware of any previous study summarizing the results of previous research investigating the reliability of PCOS diagnosis using ICD codes compared to other diagnostic criteria. We are also unaware of any previous studies investigating how many women classed as PCOS using the ICD codes have the variables required to make an objective diagnosis using the Rotterdam, NIH and AEPCOS criteria. The studies included in our scoping review also encompassed a wide range of geographical areas and ethnic groups.

On the other hand, our study found a small number of studies available that may not accurately represent the populations and data. Variations in diagnostic criteria among studies also made it difficult to draw definitive conclusions about the reliability of EHRs in diagnosing PCOS. Potential biases, also include limitations in the language of the included studies to the English language, may have restricted the number of studies identified. Ideally free androgen index and DHEAS should be tested in women with a suspected diagnosis of PCOS, but we did not collect data on these. However, this is probably not the case in routine clinical practice as reflected in our findings where SHBG was recorded in the database in 6.4% of women and reflected in the controversies surrounding the androgen measurements required in the Rotterdam criteria of PCOS [4]. A recent (2024) publication also concluded that DHEAS was not required in the diagnosis of PCOS [32]. Other limitations included our inability to perform detailed analysis by menopausal status or age group. For example, we did not investigate the detailed electronic health record of each patient to confirm their menopausal status, based on the required tests to confirm menopause, or to confirm which combination of variables (including the use of polycystic ovary morphology) was used in adolescents to reach a diagnosis of PCOS or not, when they were seen in the hospital. Our aim at this point was to investigate whether doing this would be feasible. We focused on assessing whether women in the database, all of whom had an ICD diagnosis of PCOS, possessed the necessary diagnostic variables to meet the objective criteria for diagnosing PCOS.

## 5. Conclusions

In conclusion, while the use of EHRs offers a promising and cost-effective alternative for gathering statistics on PCOS prevalence, comorbidities, and long-term health risks in large populations, our findings suggest potential underestimation of prevalence compared to other diagnostic criteria. A large portion of the women identified as having PCOS based on EHRs may also not meet the complete diagnostic criteria. These findings have significant implications for future research studies on the prevalence, comorbidities, and long-term health risks of PCOS, where EHRs alone will be used in defining PCOS. First, more specific investigations should be conducted in daily clinical practice, to ensure accurate diagnosis and avoid misclassification. Second, healthcare providers should be cautious when identifying and treating PCOS patients solely based on EHR data, as a significant portion of women identified through EHRs may not meet the complete diagnostic criteria for PCOS. Clinicians should consider additional assessments, such as the clinical history, laboratory, and ultrasound findings, to confirm diagnosis and provide appropriate treatment. Furthermore, when using EHR data for public health planning, caution should be exercised due to the limitations in accurately identifying PCOS cases. This is particularly important when determining resource allocation or designing interventions related to PCOS. Last, future research studies on PCOS should consider the potential bias introduced by relying solely on EHR data. Researchers should consider incorporating more comprehensive diagnostic criteria, combining EHR data with additional sources (such as self-reported symptoms or clinical evaluations), and conducting validation studies to ensure accurate and reliable data collection.

Future research should also focus on addressing the challenges and limitations identified in this study. This includes further studies to evaluate the accuracy and reliability of EHRs codes in diagnosing PCOS compared to other diagnostic criteria studies in diverse populations and geographical areas, to ensure the generalizability of our findings. Furthermore, studies could also explore the impact of recent updates in diagnostic criteria, such as the 2018 guidelines and the proposed FIGO classification on the feasibility and reliability of using EHRs for PCOS diagnosis in epidemiological studies. Future research should aim to overcome any language limitations in literature reviews and include studies published in languages other than English. Additionally, investigations into the sources of error in the coding process and potential improvements are needed, as adequate training and education regarding coding guidelines and practices might minimize errors and improve the overall reliability of EHRs, particularly in the context of PCOS.

However, this study is an important addition to the current scientific body of literature, as it provides research groups globally with an indication of the reliability of the prevalence estimates of PCOS acquired using EHRs, for public health and government initiatives, to improve the health and wellbeing of women with PCOS living in that society. These include measures to reduce the currently associated long term health risks of PCOS, such as diabetes, endometrial cancer, cardiovascular disease, and psychological disorders. This is important as the economic and overall wellbeing of a society depends on the health and wellbeing of the women and children living in that society.

## Figures and Tables

**Table 1:** Characteristics of studies included in the scoping review.

First Author, Year of Publication and Country or Region.	Study Design	Sample Size; Participants.	Age Groups	Definition of PCOS	Prevalence of PCOS	Accuracy of HER Diagnosis of PCOS Checked?
Lo 2006, USA [21].	Cross sectional	12,734	15–44 years	ICD-9	2.2–2.7% depending on age group	No
Okoroh 2012, USA [19]	Cross sectional	12,171,830	18–45 years	ICD-9 codes were used to create 4 mutually exclusive PCOS phenotypes that were based on the NIH, Rotterdam, and Androgen Society criteria.	1.58%	No
Christensen 2013, USA [10].	Cross sectional	137,502	15–19 years	ICD-9	0.56%	Yes
Al Khaduri 2014, Oman [20].	Cross sectional	3644	12–45 years	Rotterdam criteria	2.80%	No
Miazgowski 2019, Europe [22].	Retrospective observational	Not provided	Not provided	ICD-9 & ICD-10	0.27%	No
Sirmans 2014, USA [23] in [24]	Cross-sectional	259,904	15–45 years	ICD-9	0.88%	No
Actkins 2020, USA [18].	Cross sectional	704,970	11–44 years	ICD-9 & ICD-10 and presence of a PCOS-related keyword	5.80%	Yes
Mirza 2023, UAE [16].	Cross sectional	64,722	15–45 years	ICD-10	1.60%	No

**Table 2:** Number and proportion of the 1031 women classed as PCOD with the corresponding ICD code descriptor [16].

ICD Code Descriptor	Number (%)
H/O polycystic ovarian syndrome [Z87.42]	4 (0.4)
History of PCOS [Z87.42]	64 (6.2)
History of polycystic ovarian disease [Z87.42]	1 (0.1)
History of polycystic ovarian syndrome [Z87.42]	1 (0.1)
History of polycystic ovaries [Z87.42]	3 (0.3)
PCO (polycystic ovaries) [E28.2]	211 (20.5)
Bilateral polycystic ovarian syndrome [E28.2]	1 (0.1)
PCOD (polycystic ovarian disease) [E28.2]	82 (8)
PCOS (polycystic ovarian syndrome) [E28.2]	380 (36.9)
Polycystic disease, ovaries [E28.2]	102 (9.9)
Polycystic ovarian disease [E28.2]	45 (4.4)
Polycystic ovarian syndrome [E28.2]	28 (2.7)
Polycystic ovaries [E28.2]	79 (7.7)
Polycystic ovary disease [E28.2]	2 (0.2)
Polycystic ovary syndrome [E28.2]	6 (0.6)
Polycystic ovary [E28.2]	22 (2.1)
Total	1031

**Table 3:** Distribution of women with diagnostic variables for PCOS recorded in the database, by age groups.

Diagnostic Variable	16–18 Years (*n* = 39)	≥19 Years (*n* = 774)	*p*-Value
Serum testosterone	7 (17.9%)	218 (22%)	0.359
Sex hormone binding globulin	1 (2.6%)	65 (6.6%)	0.272
Luteinizing hormone	27 (69.2%)	718 (72.4%)	0.393
Prolactin	26 (66.7%)	695 (70.1%	0.384
Thyroid function test	32 (82.1%	860 (86.7%)	0.265
OH_17_progesterone	10 (25.6%	171 (17.2%	0.129
Follicle stimulating hormone	27 (69.2%)	730 (73.6%)	0.330
Pelvic ultrasound	35 (89.7%)	855 (86.2%)	0.364

## Data Availability

The data presented in this study for the database analysis are available on request from the corresponding author. The data are not publicly available due to the need for them to remain linked to anonymized medical record numbers for potential future linkage studies.
